# Food Insecurity was Associated With Poorer Sleep Quality and Increased Stress Among Dental Students at a HBCU and a Predominantly White School

**DOI:** 10.1002/jdd.70067

**Published:** 2025-10-20

**Authors:** Brian Laurence, Teresa A. Marshall, Fang Qian, Gillian Robinson‐Warner, Archana Kumar, Nidhi Handoo, Cari Anderson

**Affiliations:** ^1^ Department of Comprehensive Care College of Dentistry, Howard University Washington, DC USA; ^2^ Department of Preventive and Community Dentistry College of Dentistry, University of Iowa Iowa City Iowa USA; ^3^ Iowa Institute for Oral Health Research College of Dentistry, University of Iowa Iowa City Iowa USA; ^4^ Restorative Dentistry College of Dentistry, Howard University Washington, DC USA; ^5^ Department of Oral Pathology and Radiology College of Dentistry, University of Iowa Iowa City Iowa USA; ^6^ College of Dentistry, University of Iowa Iowa City Iowa USA

**Keywords:** dental students, food insecurity, HBCU, sleep disorders, stress

## Abstract

**Purpose:**

The prevalence of food insecurity (FI) and its associated outcomes among undergraduate students have been widely researched, however studies focusing on FI among professional students are limited. This study investigated the associations between FI, sleep‐related variables, and stress among dental students from predominantly Black and White dental schools in the United States during the COVID‐19 pandemic.

**Methods:**

Participants included dental students enrolled at a Historically Black College and University (Howard) and a predominately White (Iowa) dental school in the Fall of 2021. Five sleep‐related and stress outcomes were measured as dichotomous variables. The associations between FI and the outcome variables were assessed using bivariate and multivariable logistic regression analyses.

**Results:**

Mean age of the participants was 25.8 ± 3.3 years; 46% reported being food insecure. In a comprehensive multivariable model that included both Howard and Iowa students, FI was a significant (*p* < 0.05) predictor of poorer sleep quality and more frequent troublesome sleep. In stratified bivariate analyses, FI was also strongly (*p* < 0.05) associated with adverse sleep outcomes at both Howard and Iowa dental schools. Students attending Howard had higher odds of frequent troublesome sleep compared to students attending Iowa (OR = 2.92, 95% CI: 1.17–7.28). Furthermore, FI was significantly (*p* < 0.05) associated with higher stress levels across all analyses.

**Conclusions:**

Our findings are consistent with prior studies reporting associations between FI and poorer sleep outcomes and increased stress in both Black and White dental students.

## 1 Introduction

Food insecurity (FI), the condition assessed in the food security survey and represented in USDA (United States Department of Agriculture) food security reports, is defined as a household level economic and social condition of limited or uncertain access to adequate food [1]. A review of the literature has identified various adverse outcomes associated with FI among undergraduate university students including but not limited to poorer eating habits, increased stress, increased depression, lower sleep quality, poorer academic performance and increased substance abuse [2, 3, 4]. While the prevalence of FI among White undergraduate students has been more widely researched, published studies to date examining FI among Black college students are very limited [5]. Duke et al. [6] examined the prevalence of FI among students attending Historically Black Colleges and Universities (HBCUs) in the Southeastern United States (US) and found that nearly three in four students (72.9%) reported some level of FI in the past year. Compared to being a freshman, being a senior was associated with significantly increased odds of worrying that food would run out before having a chance to buy more. The authors concluded that students attending HBCUs experience FI at levels that exceed estimates reported among students attending predominantly White institutions [6].

In addition to a lack of research on FI and its associated outcomes among Black students, there is also little published research to date on FI among graduate and professional students [7, 8, 9]. Marshall et al. [10] observed that 46.9% of dental students at a Midwestern University were food insecure and dental students that were food insecure were more likely to experience food‐related stress and sleep difficulties compared to their food secure classmates. Sackey et al. [8] found that 28.5% of graduate and professional students at a Northeastern University were food insecure and that students receiving loans had a higher likelihood of being food insecure. In a cross‐sectional study of students attending 10 universities in the California system, Martinez et al. [11] observed that FI was associated with elevated body mass index (BMI). Potential mediators of this relationship included inadequate sleep and participation in fewer days of moderate‐to‐vigorous physical activity. The authors concluded that FI was associated with fewer days of enough sleep, which could possibly lead to an increased BMI and worsening health. As Duke et al. [6] summarized in their paper on FI at HBCUs, this is particularly relevant for Black students as poor dietary quality and insufficient sleep are factors that contribute to a higher prevalence of cardiometabolic diseases that occur at a higher prevalence in Blacks. In a systematic review of the literature on the association between diet and sleep quality, Godos et al. [12] concluded that despite a consistency of studies observing consumption of healthy foods was associated with better sleep quality, the overall poor‐to‐fair quality of study designs did not allow the authors to conclude that there was a causal relationship between diet and sleep quality. Most recently, Alhasan et al. [13] examined the association between FI and sleep health using National Health Interview Survey data. In the final multivariable analysis, the authors observed that FI was associated with a higher prevalence of very short sleep duration and trouble falling asleep in an ethnically diverse US sample. In another recent study that examined the associations between FI and behavioral and mental outcomes in college students, Slotnick et al. [14] concluded that FI students had a higher likelihood of having poor sleep quality and a higher likelihood of having anxiety and depression.

To our understanding, no prior studies have examined the association between FI and well‐being outcomes among dental students at both predominantly Black and White dental schools. HBCUs provide a unique opportunity to investigate this association in a predominantly Black cohort. HBCUs were established in Southern states during the late 1800's to offer Black students educational opportunities. At the time most schools of higher education did not accept Blacks; today HBCUs continue to serve predominantly. Black students and there are currently only two HBCU dental schools that operate in the US Dental students are typically older and independent of family support when compared to US undergraduate students. Dental students typically do not have significant incomes, live off campus, take on a heavy financial burden to finance their dental education, and do not participate in campus dining programs. As most previous research has been conducted in mostly white undergraduate students, the relevance of previous results to the next level of education is unknown. The rationale for addressing the gap is based on the hypothesis that if wellbeing is adversely impacted by FI, then FI presents a modifiable barrier to academic success and career potential for both Black and White students.

Previously, we examined the associations between FI status and diet quality among students in two ethnically different dental schools and observed that FI was associated with lower intakes of fruits and vegetables, higher intakes of added sugars and lower diet quality [9]. In addition, students with FI were more likely to be older, Black or other, first‐generation professional students and receiving financial assistance. In that analysis, we observed a prevalence of FI of 61.3% among students at an HBCU compared to 31.3% among students at a predominantly White dental school [9]. Given the lack of research into FI and clinical outcomes among Black college students and the historical health disparities between Black and White students, this analysis aimed to investigate associations between FI, sleep‐related variables, academic performance, and stress among dental students from predominantly Black and White dental schools in the US during the COVID‐19 pandemic.

## 2 Methods

### 2.1 Participants

Participants were students enrolled at the Colleges of Dentistry at Howard University (HBCU) (*n* = 286) and the University of Iowa (White) (*n* = 326). Howard University is located in metropolitan Washington, DC and serves a mostly Black student population, while Iowa is located in the rural Midwest and serves a mostly White student population. Details about the study procedures and sample have previously been reported [9]. All enrolled dental students in the Fall of 2021 were invited to participate in a cross‐sectional survey assessing food security status, diet‐quality, and overall well‐being. Inclusion criteria was first through fourth year dental student status; there were no exclusion criteria. The study received IRB approval from both Howard University (IRB#2021‐0151) on October 21, 2021 and the University of Iowa School (IRB#202102374) on November 2, 2021. The institutional review boards at both schools approved the study protocol; participants provided consent through survey submission using the online platform. The manuscript was prepared in accordance with STROBE Guidelines for cross‐sectional studies [15].

### 2.2 Survey Development, Independent, and Dependent Variables

The survey was designed to assess demographic characteristics, food security status, diet quality, and overall well‐being. The usual and customary demographic characteristics (i.e., age, gender, marital status, school year) were taken from existing questionnaires and non‐traditional demographic characteristics including student loan status and student generational status were modeled after questions found in the literature [3, 16]. Demographic characteristics unique to this study (i.e., annual student loans, weekly food budget, barriers to preferred diet) were developed using an iterative process and served as covariates, while the independent variable was FI measured using the validated USDA 10‐item Adult Food Security Module [10, 17]. Students were classified as food secure if they answered “no” to all questions and food insecure if they answered “yes” to one or more questions [18]. Overall diet quality was evaluated using the Short Healthy Eating Index (sHEI) previously validated in college‐aged students [19]. Wellbeing was assessed through self‐reported measures of sleep, academic performance, and stress including how worries about food or hunger impacted their sleep, academic performance, and stress [10]. The dependent variables were: 1) the likelihood of having poor or terrible sleep, categorized as “Poor” versus “Good”; 2) the likelihood of having 6 h or less sleep per night, categorized as “≤ 6 h” versus “ > 6 h”; 3) the likelihood of having trouble sleeping because of worries about food or hunger, categorized as “Yes” versus “No”; 4) the likelihood of having a very high or high stress level, categorized as “High” versus “Low”; and 5) the likelihood of sometimes to always reporting feeling stressed because of worries about food or hunger, categorized as “Yes” versus “No”.

Students were recruited by email; an initial email invitation explaining the survey was followed by two reminders at weekly intervals. The Qualtrics survey link was embedded within the email. Participation was voluntary and students provided their consent via submission of the survey which was open for 3 weeks in November 2021. No additional advertisements were used. Students were provided compensation for study participation through a $15 gift card.

### 2.3 Analytic Plan

This study employed a cross‐sectional analysis of survey data. Descriptive statistics were used to summarize the characteristics of survey respondents. Categorical variables were presented as frequencies and percentages, while continuous variables were reported as means, standard deviation, median, and ranges. Bivariate analyses, including Pearson's Chi‐square test or the nonparametric Mann–Whitney *U* test, as appropriate, were performed to identify associations between food security status and sociodemographic factors. To examine the relationship between FI and dependent variables, initial analyses were conducted using simple logistic regression. The final multivariable logistic regression models, adjusted for demographic covariates, were developed using forward logistic regression analysis and verified with backward elimination procedure. Variables with associations to the outcomes (*p* ≤ 0.10) in both bivariate and simple logistic regression analyses were included to build the final multivariable models. Odds ratios (OR) and 95% confidence intervals (95% CI) were calculated, and model fit was assessed using the Hosmer–Lemeshow test. A significance level of 0.05 was applied for all statistical tests. The analysis was conducted using SAS System version 9.4 (SAS Institute Inc., Cary, NC, USA).

## 3 Results

Table 1 shows the characteristics of the student participants and their association with FI status. Response rates were similar at Howard (32.5%) and at Iowa (29.4%). Of the 189 respondents surveyed, 68.8% identified as female, and 49.2% were Howard students. The mean age of the respondents was 25.8 ± 3.3 years, and 46% reported experiencing FI. The bivariate analysis/simple logistic regression analyses showed that 11 out of 15 selected characteristics of dental students were significantly associated with FI (*p* < 0.05 in each instance). In comparison to food secure dental students, those who were food insecure were more likely to be older, in the first dental school year, non‐White, first‐generation professional graduate students, recipients of financial aid, and have an annual student loan amount of $70,000 or more. In addition, students with FI tended to have higher average weekly food budgets, experience a COVID related impact on their ability to purchase adequate and appropriate food, be Howard dental students, and have lower average sHEI score. However, they were less likely to receive the family or spousal financial support.

**TABLE 1:** Characteristics of dental students and their association with food security status.

		Food security status		
Characteristic	All students (*N* = 189)	Food secure students (*n* = 102)	Food insecure students (*n* = 87)	*p* value^a^
**Age (years)**				**0.049** ^*^
Mean ± SD Median (Range)	25.8 ± 3.3 25 (22–39)	25.4 ± 3.1 25 (22–36)	26.3 ± 3.5 25 (22–39)	
**Dental school year (%)**				**0.008** ^*^
First Second Third Fourth	30.7 28.6 23.8 16.9	24.5 27.4 25.5 22.6	37.9 29.9 21.8 10.4	
**Gender (%)**				0.960
Male Female	31.2 68.8	31.4 68.6	31.0 69.0	
**Race (%)**				**0.016** ^*^
Caucasian Black or African American Asian Other	44.3 33.5 10.8 11.4	56.6 22.2 13.1 8.1	30.3 46.5 8.1 15.1	
**Race (%)**				**< 0.001** ^*^
Caucasian Non‐Caucasian	44.3 55.7	56.6 43.4	30.3 69.7	
**Ethnicity (%)**				0.959
Spanish or Hispanic or Latino None of these	7.9 92.1	7.8 92.2	8.1 91.9	
**Marital status (%)**				0.399
Married Never married or divorced	17.5 82.5	19.6 80.4	14.9 85.1	
**Dependent children (%)**				0.148
Yes No	7.5 92.5	4.9 95.1	10.5 89.5	
**First generation professional graduate student (%)**				**0.029** ^*^
Yes No	63.0 37.0	55.9 44.1	71.3 28.7	
**Financial aid (%)**				**0.030** ^*^
Yes No	84.7 15.3	79.4 20.6	90.8 9.2	
**Family/spousal support (%)**				**< 0.001** ^*^
Yes No	40.2 59.8	52.9 47.1	25.3 74.7	
**Annual student loan amount (%)**				**0.036** ^*^
< $10,000 $10,000–$70,000 $70,000–$90,000 > $90,000	14.5 33.9 36.0 15.6	19.8 34.6 31.7 13.9	8.2 32.9 41.2 17.7	
**Food budget, weekly (dollars)**				**< 0.001** ^*^
Mean ± SD Median (range)	100.3 ± 74.5 80 (20–600)	92.1 ± 74. 75 (20–600)	109.1 ± 74.0 100 (30–450)	
**Covid‐19 impact on purchasing adequate and appropriate food (%)**				**<** **0.001** ^*^
Yes No	20.7 79.3	8.3 91.7	35.9 64.1	
**Site (%)**				**< 0.001** ^*^
Howard Iowa	49.2 50.7	35.3 64.7	65.5 34.5	
**Diet Quality Score (sHEI Score** ^b^)				**0.003** ^*^
Mean ± SD Median (range)	47.3 ± 10.2 48.3 (24.3–67.5)	49.4 ± 10.1 50.3 (24.3–67.5)	44.9 ± 9.9 46.4 (24.6–67.2	

^a^ All *p* values are calculated based on all non‐missing responses using Chi‐square test and Mann–Whitney *U* test.

^b^ Short Healthy Eating Index score.

* Statistically significant (*p* < 0.05).

Table 2 provides a comprehensive overview of the bivariate analysis results, comparing the well‐being outcomes between food‐secure and food‐insecure dental students. Students with FI were more likely than their counterparts with food security to report poor or terrible sleep (OR = 2.92, 95% CI: 1.50–5.64; *p* = 0.002), have 6 h or fewer hours of sleep (OR = 2.19, 95% CI: 1.20–3.98; *p* = 0.011), experience frequent trouble sleeping due to worries of food or hunger (OR = 4.68, 95% CI: 2.12–10.32; *p* < 0.001), report very high or high stress level (OR = 2.60, 95% CI: 1.32–5.10; *p* = 0.006), and sometimes or always feel stressed because of worries about food or hunger (OR = 7.16, 95% CI: 3.43–14.94; *p* < 0.001). Stratified bivariate analysis by institution revealed evidence of interaction. Several associations between FI and well‐being outcomes differed significantly depending on whether students were from the Howard or Iowa (Tables S1 and S2). At Iowa, students with FI were 6.39 times as likely to experience frequent trouble sleeping than students with food security (OR = 6.39, 95% CI: 1.52–26.82; *p* = 0.009), compared to 2.68 times as likely at Howard (OR = 2.68, 95% CI: 1.00–7.17; *p* = 0.046). Similarly, students with FI at Iowa were 8.13 times as likely to feel stressed because of worries about food or hunger (OR = 8.13, 95% CI: 2.53–26.16; *p* < 0.001), compared to 4.78 times as likely at Howard (OR = 4.78, 95% CI: 1.80–12.72; *p* = 0.001).

**TABLE 2:** Bivariate associations of dental students’ well‐being and academic performance with food insecurity^a^.

		Food security status			
Variable	Total (*N* = 189)	Food Insecure (*N* = 87)	Food Secure (*N* = 102)	Unadjusted OR (CI)^b^	*p* value
**Sleep quality (%)**					**0.002** ^*^
Poor or terrible Excellent and very good or good	28.3 71.7	40.0 60.0	18.6 81.4	2.92 (1.50–5.64) 1.00	
**Average hours of sleep at night (%)**					**0.011** ^*^
6 h or less 7 h or more	58.5 41.5	68.6 31.4	50.0 50.0	2.19 (1.20–3.98) 1.00	
**Frequency of trouble sleeping because of worries about food or hunger (%)**					**< 0.001** ^*^
Sometimes to always Never	20.7 79.3	33.7 66.3	9.8 90.2	4.68 (2.12–10.32) 1.00	
**GPA** ^c^ **(%)**					**0.008** ^*^
Less than 3.5 3.5–4.0	62.6 37.4	72.9 27.1	53.9 46.1	2.30 (1.24–4.27) 1.00	
**Frequency of worries about food or hunger negatively affecting academic performance (%)**					**< 0.001** ^*^
Sometimes to always Never	34.6 65.4	52.3 47.7	19.6 80.4	4.50 (2.36–8.59) 1.00	
**Stress level**					**0.006** ^*^
Very high or high Expected/low/limited	71.3 28.7	81.4 18.6	62.7 37.3	2.60 (1.32–5.10) 1.00	
**Frequency of feeling stressed because of worries about food or hunger**					**< 0.001** ^*^
Sometimes to always Never	28.7 71.3	48.8 51.2	11.8 88.2	7.16 (3.43–14.94) 1.00	

^a^ Results of simple logistic regression with food insecurity status, and each row of the table represents a separate model.

^b^ OR, odds ratio; CI, 95% Wald Confidence Limits; “1.00”, reference category.

^c^ Grade point average

* *p* < 0.05.

Table 3 shows the multivariable analysis results for the well‐being outcomes.

**TABLE 3:** Significant predictors of participant characteristics for six well‐being outcomes analyzed using stepwise logistic regression^a^, ^b^ (final model, giving odds ratio adjusted for other variables in the model).

Variables	Sleep quality (poor vs. good) OR (CI)^c^	Average hours of sleep (≤ 6 vs. ≥ 7 h) OR (CI)^c^	Frequency of trouble sleeping (yes vs. no) OR (CI)^c^	Frequency of worries about food or hunger (Yes vs. No) OR (CI)^c^	Stress level (high vs. low) OR (CI)^c^	Frequency of feeling stressed (yes vs. no) OR (CI)^c^
**Food security status**					
Insecure Secure	2.89 (1.34–6.20)^*^ 1.00	—^e^	2.64 (1.01–6.89)^*^ 1.00	2.98 (1.34–6.62)^*^ 1.00	2.46 (1.18–5.13)^*^ 1.00	5.19 (2.18–12.39)^*^ 1.00
**Site (%)**					
Howard Iowa	—	15.50 (6.21–38.71)^*^ 1.00	2.92 (1.17–7.28)^*^ 1.00	2.87 (1.31–6.29)^*^ 1.00	**—**	2.38 (1.03–5.48)^*^ 1.00
**Age (years)**					
	—	–	—	—	**—**	**—**
**Dental school year**					
First Second Fourth Third	–	6.28 (2.07–19.01)^*^ 2.58 (0.89–7.51) 2.04 (0.61–6.84) 1.00	—	—	**—**	**—**
**Gender**					
Female Male	—	—	—	—	3.06 (1.50–6.23)^*^ 1.00	—
**Ethnicity**					
Spanish or Hispanic or Latino None of these	5.17 (1.50–17.77)^*^ 1.00	—	—	0.05 (0.01–0.52)^*^ 1.00	—	0.06 (0.01–0.63)^*^ 1.00
**Marital status**					
Married Never married or Divorced	—	—	—	—	—	—
**Dependent children**					
Yes No	—	—	—	—	—	—
**First generation professional graduate student**					
Yes No	—	3.10 (1.13–8.51)^*^ 1.00	—	2.71 (1.02–7.17)^*^ 1.00	**—**	**—**
**Financial aid**					
Yes No	—	—	—	—	**—**	**—**
**Family/spousal support**					
No Yes	—	—	3.55 (1.20–10.51)^*^ 1.00	—	**—**	**—**
**Annual student loan amount**					
> $90,000 $70,000–$90,000 $10,000–$70,000 < $10,000	5.45 (1.25–23.64)^*^ 1.41 (0.35–5.77) 2.45 (0.62–9.66) 1.00	—	—	—	**—**	**—**
**Food budget, weekly (dollars)**					
	1.%2 (1.00–1.01)^*^	—	—	—	**—**	**—**
**Covid‐19 impact on purchasing adequate and appropriate food**					
Yes No	—	3.09 (1.08–8.85)^*^ 1.00	3.48 (1.39–8.74)^*^ 1.00	5.51 (1.97–15.41)^*^ 1.00	**—**	6.44 (2.35–17.66)^*^ 1.00
**Diet Quality Score (sHEI Score** ^d^ **)**					
	—	—	—	**—**	0.96 (0.93–1.00)^*^	**—**

^a^ Each column (2–7) of the table represents a separate model using a logistic regression model adjusting for food security status and school site and along with selected sociodemographic variables.

^b^ Variables with a *p* value of 0.10 or less in the bivariate analysis were admitted into the final stepwise logistic regression procedure.

^c^ Odds ratio (Confidence Interval).

^d^ Short Healthy Eating Index.

^e^ “ — ” Statistically nonsignificant in each stepwise logistic regression model.

* Statistically significant (*p* < 0.05).

### 3.1 Sleep Quality

The final stepwise multivariable logistic regression analysis for sleep quality revealed that FI status, ethnicity, the amount of annual student loan, and weekly food budget as significant predictors of poor sleep quality. Dental students with FI had significantly higher odds of reporting poor sleep quality compared to their counterparts with food security (OR = 2.89, 95% CI: 1.34–6.20; *p* = 0.007). Similarly, students identifying as Spanish, Hispanic, or Latino exhibited were more likely to report poor sleep compared to those not in this ethnic category (OR = 5.17; 95% CI: 1.50–17.77; *p* = 0.009). A higher weekly average food budget was also associated with an increased likelihood of poor sleep (OR = 1.01, 95% CI: 1.00–1.01; *p* = 0.031). In addition, students with an annual student loan exceeding $90,000 were at greater risk of poor sleep compared to those with loans under $10,000 (OR = 5.45, 95% CI: 1.25–23.64; *p* = 0.024).

### 3.2 Average Hours of Sleep at Night

Significant predictors for reduced average hours of sleep at night (≤ 6 h per night) included school site, year in dental school, first‐generation professional graduate status, and the impact of COVID on purchasing adequate food. Howard dental students exhibited higher odds of sleeping less than or equal to 6 h per night compared to Iowa dental students (OR = 15.50, 95% CI: 6.21–38.71; *p* < 0.001). Furthermore, first‐year dental students had higher odds of having less than or equal to 6 h of sleep at night compared to third‐year dental students (OR = 6.28, 95% CI: 2.07–19.01; *p* = 0.004). First‐generation professional graduate students were more likely to experience reduced sleep at night than those who were not (OR = 3.10, 95% CI: 1.13–8.51; *p* = 0.028). Those who perceived an impact of COVID‐19 on purchasing adequate and appropriate food were also more likely to have less than or equal to 6 h of sleep compared to those who did not perceive such an impact (OR = 3.09, 95% CI: 1.08–8.85; *p* = 0.035). However, FI status was not significantly associated with reduced sleep duration.

### 3.3 Frequency of Trouble Sleeping

Significant predictors for frequent trouble sleeping included FI status, school site, family or spousal financial support, and the perceived impact of COVID‐19 on purchasing adequate and appropriate food. Howard dental students exhibited higher odds of experiencing frequent trouble sleeping compared to Iowa dental students (OR = 2.92, 95% CI: 1.17–7.28; *p* = 0.021). In addition, students with FI had increased odds of frequent trouble sleeping compared to their counterparts with food security (OR = 2.64, 95% CI: 1.01–6.89; *p* = 0.048).

### 3.4 Stress Level

Predictors of higher stress levels included FI status, gender, and diet quality score (sHEI). Students with FI had higher odds of experiencing high stress compared to students with food security (OR = 2.46, 95% CI: 1.18–5.13; *p* = 0.017). Female dental students were more likely to have higher stress than male dental students (OR = 3.06, 95% CI: 1.50–6.23; *p* = 0.002). Furthermore, for every 1 unit increase in diet quality score, the OR of having high stress decreased by 4% (OR = 0.96, 95% CI: 0.93–0.99; *p* = 0.037).

### 3.5 Feeling Stressed Because of Worries About Food or Hunger

FI status, school site, ethnicity, and the impact of COVID on purchasing adequate food were significant predictors of stress related to worries about food or hunger. In comparison to Iowa dental students, Howard dental students exhibited higher odds of feeling stressed due to worries about food or hunger (OR = 2.38, 95% CI: 1.03–5.48; *p* = 0.042). Students with FI had increased odds of feeling stressed due to worries about food or hunger compared with their counterparts with food security (OR = 5.19, 95% CI: 2.18–12.38; *p* < 0.001).

## 4 Discussion

FI was a significant predictor of poorer sleep quality and more frequent troublesome sleep in a comprehensive multivariable model that included both Black and White students. In stratified bivariate analyses, FI was also strongly associated with poorer sleep outcomes at both an HBCU Dental School (Howard) and a predominantly White Dental School (Iowa). The OR for more frequent troublesome sleep at Iowa was more than twice as large as that observed at Howard. We also observed similar outcomes in our analyses of the association between FI and stress.

Our results are largely consistent with prior studies that have reported FI to be associated with worsening sleep outcomes among adults [4, 11, 12, 13, 15, 20]. However, our results expand on earlier works by focusing on Black dental students in addition to a predominantly White dental student population. As discussed by Martinez et al. [11] in their paper, sleep sufficiency represents an important pathway linking FI to BMI and overall health and this is especially important among Black adults who have a higher prevalence of both BMI and adverse overall health outcomes. In addition, FI is associated with worse dietary outcomes and poorer dietary quality, making it a proxy measure for poor dietary outcomes among adults [21, 22]. There are several plausible pathways through which FI or a poor diet can lead to worsening sleep health. A comprehensive discussion of these pathways is described in the systematic review of the association between diet and sleep quality by Godos et al. [12]. Briefly, one path is that uncertainty about where the next meal will come from, choices between paying the rent or buying food, or other food‐related stresses increase psychological distress which can activate the sympathoadrenal medullary system and hypothalamic–pituitary–adrenal (HPA) axis which have been linked to poor sleep health [13, 23]. Another pathway may be through an increase in the glycemic index as a result of a less healthy diet which could lead to shortened sleep onset and increased difficulty in obtaining restful sleep [12]. In addition, FI is also a proxy for a poorer diet that can lead to an increased inflammatory response and the increased levels of subclinical inflammation could lead to sleep disturbances [24]. Finally, FI may lead to worsening sleep directly through a restrictive caloric intake due to hunger directly affecting sleep quality [13].

Our results are also consistent with other studies that have found associations between FI and worsening mental health outcomes including an increased prevalence of stress and depressive symptomatology [25, 26, 27]. We observed the associations between FI and increased stress separately at both Howard and Iowa dental schools and also in the multivariable model that included the sociodemographic variables. Again, our study is one of the first to examine the association between FI and mental health among both Black and White dental students and the magnitude of the associations in both bivariate and multivariable analysis demonstrates a need for further research including prospective studies. In a study examining the association between FI and mental health among low‐income participants during the COVID‐19 pandemic, Fang et al. [26] observed that the OR of anxiety from being food insecure was 1.91 (95% CI: 1.53–2.39), similar in magnitude to the association that FI had on stress level among dental students during COVID‐19 in our final multivariable model (OR = 2.46, 95% CI: 1.18–5.13). There was a significant increase in stress and anxiety levels among graduate and undergraduate students during the COVID‐19 pandemic and a lack of food or limited access to food is one of the multiple pathways that the pandemic led to worsening mental health [28].

The adverse health outcomes associated with being food insecure at both Howard and Iowa has consequences that reach beyond the short‐term outcomes we have described so far. Food insecure students are less likely to graduate which means that FI may be having an impact on how many Black dentists are being produced, many of whom choose to practice in underserved populations. This study occurred during the ending of the COVID‐19 pandemic in November 2021, and it is unclear if those unique pandemic conditions still apply. Students were eligible for benefits from the Supplemental Nutrition Assistance Program (SNAP) during COVID‐19 and from the Coronavirus Aid, Relief, and Economic Security (CARES) Act and studies have shown that SNAP participation was beneficial for reducing the adverse effects of FI for college students during the COVID‐19 pandemic [29]. Many of the studies that have examined the impact of FI on students have been cross‐sectional, but given the potential long‐term impact that FI could have on the numbers of Black dentists that are produced annually, prospective studies are urgently needed that examine the long‐term effects of FI on the production of Black health care providers, not just dentists, given the important role Black health care providers play in treating the Black community. Our results are important because like the study on FI prevalence at HBCUs by Duke et al. [6], the results of our study highlight the consequences of FI among a traditionally underserved student population that could have broader implications for the underserved community in the US. Additional research is necessary to determine if addressing FI improves overall diet quality and reduces adverse academic and well‐being outcomes among Black and White dental students.

Despite the importance of our results, they should nevertheless be interpreted with caution and in light of a few limitations. These include the cross‐sectional design, the assessments for the dependent variables, self‐selection bias, and the relatively small sample size. Our measures for the dependent variables were self‐reported and not validated instruments designed to measure these outcomes under ideal conditions. Self‐selection is also a concern given that food secure students may have been less interested in participating in the survey. In addition, our limited sample size meant that more detailed exploratory analyses were not possible. Our focus on dental students limits the generalizability of our results to other health profession groups. Finally, while our subjects represent two very different student populations within the US, they are not representative of the breadth of dental schools across the country which further limits generalizability.

## 5 Conclusion

In summary, FI was associated with worsening sleep quality and increased stress levels in both Howard and Iowa dental student populations. These associations remained significant in multivariable models adjusting for sociodemographic variables. Further research should focus on exploring these relationships in prospective studies, identify strategies to alleviate the burden of FI among graduate students, and examine the long‐term career impact of FI on Black and White dental students.

## Ethics Statement

The study was separately approved by Howard University's and the University of Iowa's Institutional Review Boards.

## Conflicts of Interest

The authors declare no conflicts of interest.

## Supporting information




**Supporting File 1**: jdd70067‐sup‐0001‐tablesS1‐S2.docx
