# Supplementary material for: Food Insecurity was Associated With Poorer Sleep Quality and Increased Stress Among Dental Students at a HBCU and a Predominantly White School
Source: J Dent Educ. 2025 Oct 20;90(7):1044–53. doi: 10.1002/jdd.70067 (PMC13371973; doi:10.1002/jdd.70067)
Supplement: Supplementary file 1 — Supporting File 1: jdd70067‐sup‐0001‐tablesS1‐S2.docx [file JDD-90-1044-s001.docx]

**Supplementary Table 1. Bivariate Associations of Howard University Dental Students’ Well-being and Academic Performance with Food Insecurity^1^**

| **Variable** | **Total**  **(N=93)** | **Food Security Status** | | | |
| --- | --- | --- | --- | --- | --- |
|  |  | **Food Insecure**  **(N=57)** | **Food Secure**  **(N=36)** | **Unadjusted**  **OR (CI)^2^** | ***p-value**** |
| **Sleep Quality (%)**  Poor or Terrible  Excellent & Very good or Good | 34.1  65.9 | 43.6  56.4 | 19.4  80.6 | 3.21 (1.20-8.57)  1.00 | **0.017*** |
| **Average hours of sleep at night (%)**  6 hours or less  7 hours or more | 82.6  17.4 | 83.9  16.1 | 80.6  19.4 | 1.26 (0.42-3.75)  1.00 | 0.677 |
| **Frequency of trouble sleeping because of worries about food or hunger (%)**  Sometimes to Always  Never | 31.5  68.5 | 39.3  60.7 | 19.4  80.6 | 2.68 (1.00-7.17)  1.00 | **0.046*** |
| **GPA^3^ (%)**  Less than 3.5  3.5-4.0 | 73.6  26.4 | 78.2  21.8 | 66.7  33.3 | 1.79 (0.70-4.60)  1.00 | 0.223 |
| **Frequency of worries about food or hunger negatively affecting academic performance (%)**  Sometimes to Always  Never | 46.7  53.3 | 60.7  39.3 | 25.0  75.0 | 4.64 (1.84-11.70)  1.00 | **<0.001*** |
| **Stress Level**  Very high or High  Expected/Low/Limited | 75.0  25.0 | 80.4  19.6 | 66.7  33.3 | 2.05 (0.79-5.32)  1.00 | 0.139 |
| **Frequency of feeling stressed because of worries about food or hunger**  Sometimes to Always  Never | 40.2  59.8 | 53.6  46.4 | 19.4  80.6 | 4.78 (1.80-12.72)  1.00 | **0.001*** |

**^1^** Results of simple logistic regression with food insecurity status, and each row of the table represents a separate model.

**^2^**OR, odds ratio; CI, 95% Wald Confidence Limits; “1.00”, reference category.

^3^GPA = Grade point average

*******p* < 0.05.

**Supplementary Table 2. Bivariate Associations of University of Iowa Dental Students’ Well-being and Academic Performance with Food Insecurity^1^**

| **Variable** | **Total**  **(N=96)** | **Food Security Status** | | | |
| --- | --- | --- | --- | --- | --- |
|  |  | **Food Insecure**  **(N=30)** | **Food Secure**  **(N=66)** | **Unadjusted**  **OR (CI)^2^** | ***p-value**** |
| **Sleep Quality (%)**  Poor or Terrible  Excellent & Very good or Good | 22.9  77.1 | 33.3  66.7 | 18.2  81.8 | 2.25 (0.84-6.02)  1.00 | 0.102 |
| **Average hours of sleep at night (%)**  6 hours or less  7 hours or more | 35.4  64.6 | 40.0  60.0 | 33.3  66.7 | 1.33 (0.55-3.25)  1.00 | 0.527 |
| **Frequency of trouble sleeping because of worries about food or hunger (%)**  Sometimes to Always  Never | 10.4  89.6 | 23.3  76.7 | 4.6  95.4 | 6.39 (1.52-26.82)  1.00 | **0.009*** |
| **GPA^3^ (%)**  Less than 3.5  3.5-4.0 | 52.1  47.9 | 63.3  36.7 | 47.0  53.0 | 1.95 (0.80-4.73)  1.00 | 0.137 |
| **Frequency of worries about food or hunger negatively affecting academic performance (%)**  Sometimes to Always  Never | 22.9  77.1 | 36.7  63.6 | 16.7  83.3 | 2.89 (1.08-7.75)  1.00 | **0.031*** |
| **Stress Level**  Very high or High  Expected/Low/Limited | 67.7  32.3 | 83.3  16.7 | 60.6  39.4 | 3.25 (1.10-9.57)  1.00 | **0.027*** |
| **Frequency of feeling stressed because of worries about food or hunger**  Sometimes to Always  Never | 17.7  82.3 | 40.0  60.0 | 7.6  92.4 | 8.13 (2.53-26.16)  1.00 | **<0.001*** |

**^1^** Results of simple logistic regression with food insecurity status, and each row of the table represents a separate model.

**^2^**OR, odds ratio; CI, 95% Wald Confidence Limits; “1.00”, reference category.

^3^GPA = Grade point average

******p* < 0.05.
